# Epidermal growth factor receptor dimerization status determines skin toxicity to HER-kinase targeted therapies

**DOI:** 10.1038/sj.bjc.6602875

**Published:** 2005-11-22

**Authors:** I Laux, A Jain, S Singh, D B Agus

**Affiliations:** 1 Louis Warschaw Prostate Cancer Center, Cedar-Sinai Medical Center, 8631 West Third Street, Suite 1001E, Los Angeles, CA 90048, USA; 2 Monogram Biosciences, Inc., 345 Oyster Point Blvd., South San Francisco, CA 94080-1913, USA

**Keywords:** skin rash, EGFR, HER2, gefitinib, pertuzumab, keratinocytes

## Abstract

Skin toxicity, a common drug-related adverse event observed in cancer patients treated with epidermal growth factor receptor (EGFR)-directed therapies is rarely seen with therapies targeting HER2. This study reports the significance of the EGFR and HER2 dimerization status in skin with regard to these dermatologic side effects. We demonstrate the differential effect of HER-directed therapies on the ligand driven activation status of EGFR, HER2 and MAPK in normal human epidermal keratinocytes. EGFR-directed therapies, such as gefitinib and cetuximab, inhibited ligand-induced activation of EGFR and MAPK in human keratinocytes. Pertuzumab, an antibody interfering with functional HER2 heterodimerization, failed to block ligand-induced HER signaling in primary keratinocytes. Using a novel proximity-based dimerization assay (*eTag*™) we show that EGFR homodimers are the predominant HER dimer pair in normal primary kertinocytes and in normal skin tissue from 16 patients with solid malignancies. The presence of [p]EGFR and [p]MAPK, but the absence of [p]HER2, demonstrates productive signaling via EGFR but not HER2 in human skin. These data illustrate the importance of the EGFR dimerization partner in human skin and suggests that inhibition of EGFR homodimer signaling rather than EGFR/HER2 heterodimer signaling maybe the key molecular event determining dermatologic toxicity discrepancies observed between EGFR-targeted versus HER2-targeted therapies.

Members of the human epidermal receptor (HER/ErbB) family, in particular epidermal growth factor receptor (EGFR/HER1/ErbB1) and HER2/neu/ErbB2 play key roles in the tumorigenic process of epithelial cancers and have been attractive candidates for the development of target-based treatment strategies. Considerable progress in drug development targeting EGFR and/or HER2 has been made providing promising treatment options in a subset of solid malignancies (Mendelsohn and Baselga, 2000; Arteaga, 2003; Rowinsky, 2003).

Skin rash and diarrhea are the most common side effects in patients treated with EGFR-directed therapies. Cutaneous toxicities, commonly defined as rash, acne, pruritus and dry skin, have been reported as one of the predominant side effects in cancer patients treated with small-molecule tyrosine kinase inhibitors (TKI) of EGFR (gefitinib, erlotinib), dual EGFR/HER2 (lapatinib) inhibitors, pan-HER (CI-1033) inhibitors and anti-EGFR antibodies (cetuximab, ABX-EGF and EMD72000) (Busam *et al*, 2001; Hidalgo *et al*, 2001; Shin *et al*, 2001; Fukuoka *et al*, 2003; Kris *et al*, 2003; Burris, 2004; Rowinsky *et al*, 2004; Vanhoefer *et al*, 2004).

Interestingly, the characteristic cutaneous side effects observed with EGFR-directed therapies have not been reported for therapies targeting HER2, such as trastuzumab and pertuzumab (2C4, Omnitarg™). Pertuzumab is a HER2-specific humanized monoclonal antibody, which inhibits ligand-mediated intracellular signaling by blocking functional HER2 heterodimerization (Agus *et al*, 2002; Mendoza *et al*, 2002). Pertuzumab has a favorable toxicity profile and demonstrated clinical activity in a Phase I clinical trial in patients with advanced solid cancers (Agus *et al*, 2005b). Skin rash of grade 2 and greater (NCI-CTC) is dose dependent and occurs in 20–50% of patients treated with EGFR-directed therapies such as gefitinib (Fukuoka *et al*, 2003), erlotinib (Soulieres *et al*, 2004) and cetuximab (Saltz *et al*, 2004). However, only about 1% of the patients in phase I and II studies with pertuzumab developed skin rash greater than grade 1 which did not demonstrate the rash characteristics typical for EGFR inhibitor therapy (Agus *et al*, 2005a, 2005b); (S Kelsey, Genentech, personal communication).

Skin has been suggested as a surrogate tissue for pharmacodynamic endpoint evaluation for EGFR-directed therapies because it is easy assessable, expresses EGFR and frequently develops toxicity following these therapies (Baselga, 2003). Several clinical studies have shown a positive correlation of the incidence of skin rash and tumour response or survival (Baselga, 2003; Clark *et al*, 2003; Cohen *et al*, 2003; Perez-Soler, 2003; Saltz *et al*, 2003; Perez-Soler *et al*, 2004; Soulieres *et al*, 2004). However, other studies have failed to observe this correlation (Tan *et al*, 2004). Although skin rash may be a pharmacodynamic marker of drug action (Albanell *et al*, 2002; Malik *et al*, 2003), its predictive value and potential as a surrogate marker for response to EGFR-targeted agents depends on the correlation of HER-kinase signaling in paired skin and tumour tissue which requires further investigation.

Our study takes the first step towards addressing this clinical question and analyzes the molecular mechanism(s) potentially responsible for skin rash observed in patients treated with HER-kinase directed therapies.

## MATERIALS AND METHODS

### Material

RPMI 1640 and Keratinocyte Growth Medium (KGM) were obtained from ATCC and Cambrex (Walkersville, MD, USA), respectively. Antibodies were purchased as follows: EGFR (#2232), HER2 (#2242), pEGFR (#2236), pHER2 (#2249) and pMAPK (#9101) from Cell Signaling Technology (Beverly, MA, USA), HER3 (2F12) from Neomarkers (Fremont, CA, USA), *β*-actin from Sigma (St Louis, MO, USA). Secondary horseradish peroxidase-conjugated anti-rabbit and anti-mouse antibodies were obtained from Amersham Biosciences (Piscataway, NJ, USA). EGF was purchased from Invitrogen (Carlsbad, CA, USA), TGF*α* from Calbiochem (Fremont, CA, USA) and heregulin (HRG) from R&D, Minneapolis, MN, USA).

### Cell culture

Adult normal human epidermal keratinocytes (NHEK) were obtained from Cambrex (Walkersville, MD, USA) and maintained in KGM medium supplemented with growth factors, cytokines and supplements according to manufacturer's instructions. 22Rv1 cells (ATCC) or MCF7 cells (ATCC) were cultured in RPMI 1640 or DMEM/F12 containing 10% FBS, 2 mM L-glutamine and penicillin/streptomycin (100 U ml^−1^), respectively.

### Cell stimulation and lysis

NHEK were starved overnight in basal KGM medium without supplements and 22Rv1 cells were serum-starved in RPMI 1640 without phenol red supplemented with 0.02% BSA before they were treated with increasing concentrations of gefitinib (AstraZeneca, Alderley Park, Cheshire, UK) for 2 h at 37°C. Ligand stimulation was carried out using 4 nM EGF, 5 nM TGF*α* or 100 nM HRG for 10 min at 37°C before cells were lysed in RIPA lysis buffer (50 mM Tris-HCl, 1% NP-40, 0.1% SDS, 0.25% sodium deoxycholate, 150 mM NaCl, 1 mM EDTA). Protein quantitations were performed using a BCA protein quantitation kit from Pierce (Rockford, IL, USA).

### Immunoblotting and immunoprecipitation

For immunoblotting, cell lysates were heated to 100°C for 5 min in presence of Laemmli sample buffer. Equivalent amounts of protein lysates were subjected to SDS-polyacrylamide gel electrophoresis, transferred to PDVF membranes and immunoprobed with the appropriate antibodies. Blots were analyzed by chemiluminescence using HRP-conjugated secondary antibodies and ECL detection reagent (Amersham Pharmacia Biotech, Piscataway, NJ, USA).

For immunoprecipitations, cell lysates containing equivalent amounts of protein were incubated overnight at 4°C with 2 *μ*g of the appropriate antibodies. After adding 20 *μ*l of 50% slurry of protein-A Sepharose beads, cell lysates were incubated for another 2 h at 4°C. The immunoprecipitates were pelleted by centrifugation (3000 r.p.m., 4°C) and washed three times with RIPA lysis buffer to remove nonspecific proteins. The captured immunocomplexes were eluted by boiling the beads in a 2 × SDS sample buffer for 5 min. The samples were then immunoblotted as described above.

### RNA extraction and real-time quantitative RT-PCR

Total RNA was extracted from cells lines using Trizol reagent (GIBCO/BRL Life Technologies, MD, USA). DNase I (Ambion, CA, USA) was used to rid the samples of genomic DNA and total RNA yield was quantified spectrophotometrically. The RT-PCR was performed by using TAQman One-Step RT-PCR Master Mix Reagents Kit (Applied Biosystems, Foster City, CA, USA). PCR cycling conditions were as follows: 30 min at 48°C for RT step; 10 min at 95°C for AmpliTaq Gold Activation; and 40 cycles for denaturation (95°C, 15 s), and annealing/elongation (60°C for 1 min) steps. PCR reactions for each template were run in triplicate using 1 *μ*g of total RNA per sample. The sequences of the primer/probe sets for the HER-kinase axis were described previously (Agus *et al*, 2002). Standard curves were constructed for each gene-specific primer pair using 10–1000 ng of total RNA prepared from CWR22R xenografts. *β*-Actin was used as a normalization control using the following primer/probe set: (F) 5′-GCGCGGCTACAGCTTCA-3′, (R) 5′-TCTCCTTAATGTCACGCACGAT-3′, (P) 5′-FAM- CACCACGGCCGAGCGGGA-TAMRA-3′. All of the experiments were optimized such that the threshold cycle (C_T_) from triplicate reactions did not span more than one cycle number. The comparative C_T_ method (PE Applied Biosystems, Foster City, CA, USA) was used to determine relative quantification of gene expression for each gene compared with the *β*-actin control. Average C_T_ values from triplicate PCR reactions for the genes of interest were normalized to average C_T_ values for *β*-actin from the same RNA/cDNA preparation.

### Patient skin samples

Sixteen formalin-fixed paraffin-embedded (FFPE) skin tissue samples from patients with solid tumours who had previously undergone surgical procedures at Cedars-Sinai Medical Center (CSMC) were analyzed. The skin tissue was collected after informed consent was obtained from each patient. All human investigations were performed after approval by an institutional review board.

### Proximity-based *eTag*™ assays

*eTag*™ assays are multiplexed proximity-based assays that evaluate protein expression, dimerization and phosphorylation simultaneously via multi-label binding of specific antibodies to nonoverlapping epitopes on the same protein and/or interacting protein partners (Miller, 2002; Chan-Hui *et al*, 2004). HER-receptor phosphorylation was evaluated with antiphosphotyrosine antibodies and ERK phosphorylation was assessed with an antiphospho ERK1/2 antibody. The multiple antibody–analyte binding events occur in the solution phase of the lysates during incubation for 1 h at RT to overnight at 4°C. Typically, each antibody is conjugated with a unique fluorescent *eTag*™ moiety via a cleavable linker. Each *eTag*™ is distinguishable from each other upon analysis by capillary electrophoresis (CE). One specific antibody is coupled to a ‘molecular scissor’, which upon illumination by green light will emit singlet oxygen. *eTag*™ reporters that locate within a couple of hundred nanometers from the ‘molecular scissor’ are released via photo cleavage of the linker by singlet oxygen and then collected for CE analysis following a buffer exchange step to remove excess salt. The identification of each *eTag*™ reporter is determined with *eTag*™ Informer software and the quantification is based on the CE peak area normalized to an internal CE standard marker.

Cell lysates were prepared by adding 500 *μ*l of ice-cold ACLARA lysis buffer per 10 cm dish containing phosphatase and protease inhibitors. Crude extracts were microcentrifuged at 14 000 r.p.m. for 10 min at 4°C to remove insoluble materials. The total protein concentration in each lysate was determined by BCA assay (Pierce, Rockford, IL, USA). The lysates were serially diluted with lysis buffer in a 7-point titration curve ranging from 100 to 1.6 *μ*g total-protein equivalents per assay. The analysis at each titration point was performed in triplicates. Background for the *eTag*™ reporter readout was determined by omitting the cell lysates in the assay and later subtracting it from each sample. The *eTag*™ readouts in relative fluorescence units (RFU) correspond to 20 *μ*g total protein-equivalents of lysates that reside in the linear portion on the titration curves throughout all samples used for reporting.

FFPE skin sections on glass slides were analyzed with two multiplexed *eTag*™ assays. One assay was used to analyze EGFR homodimerization and EGFR phosphorylation on tyrosine residues. A second assay was used to determine HER2 phosphorylation. Receptor phosphorylation was measured using the proximity-based release of *eTag*™ reporter from conjugated antiphospho-tyrosine on the same molecules bound by scissor-coupled anti-EGFR or anti-HER2. To measure EGFR homodimerization, a single monoclonal antibody was separately coupled with either the ‘molecular scissor’ or another eTag™ reporter. Since the monoclonal antibody binds to a single epitope, the scissor and reporter will be in proximity only when EGFR forms a homodimer with itself. Approximately half of such homodimerization events will lead to the proximity-based *eTag*™ release when the scissor-anti-EGFR and *eTag*™ -anti-EGFR are mixed at 1 : 1 ratio in the assay. To normalize EGFR homodimerization, EGFR or HER2 phosphorylation readouts for cellular content in each tissue section and the variation in section size, tubulin was used as an internal control. Tubulin was quantified in the same multiplexed assay by including two nonoverlapping tubulin-specific antibodies, one conjugated with a unique *eTag*™ reporter and the other coupled with the ‘molecular scissor’.

EGFR, HER2 and HER3 expression data and phospho-EGFR and phospho-ERK data are presented as the average relative fluorescence units (RFU) of a triplicate analysis for each sample including the standard deviation. EGFR homodimerization and EGFR/HER2 heterodimerization data are presented as the ratio of homodimerized EGFR to total EGFR or heterodimerized EGFR to total HER2, respectively. These ratios are the average of a triplicate analysis for each sample. Standard deviations were calculated for each ratio.

## RESULTS

### EGFR is the major HER/ErbB receptor in primary human keratinocytes

**Figure 1:**
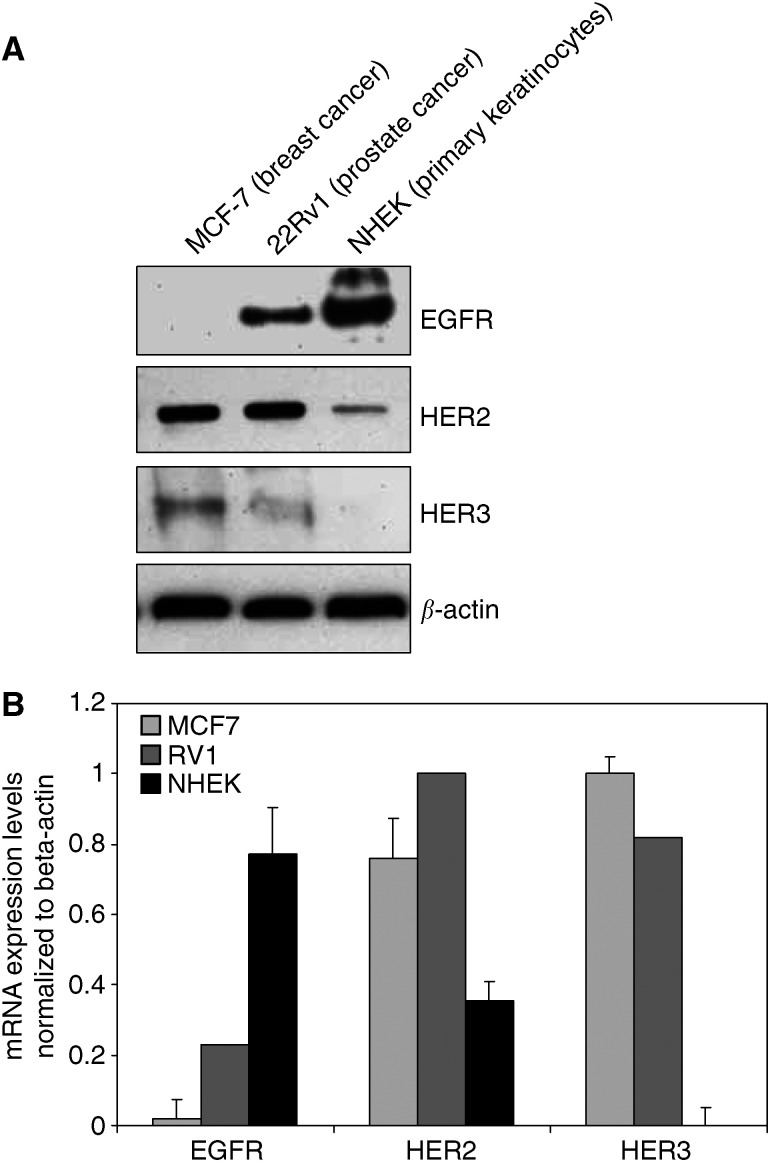
HER-kinase receptor expression in NHEK and various cancer cell lines. (**A**) Immunoblot analysis of cell lysates from NHEK and various cancer cell lines. Cells were lysed and equal amounts of total protein were subjected to SDS-PAGE and transferred to a PDVF-membrane. The blot was probed with specific antibodies to EGFR, HER2 and HER3 and reprobed for *β*-actin to account for loading differences. (**B**) Real time quantitative RT-PCR analysis of EGFR, HER2 and HER3 mRNA expression in MCF-7 cells (*light gray bars*), 22Rv1 cells (*dark gray bars*) and NHEK (*black bars*). The absolute receptor mRNA expression levels were normalized to *β*-actin mRNA expression. The data for each receptor is presented as relative receptor expression of the evaluated cell lines to each other.

To characterize the abundance of the HER-kinase receptors in primary normal human epidermal keratinocytes (NHEK), protein expression levels for EGFR, HER2 and HER3 were assessed and compared to cancer cell lines of various origins with known HER-kinase receptor expression patterns (Figure 1A). EGFR was found to be the major HER-kinase receptor expressed in human keratinocytes as previously reported by other groups (Rodeck *et al*, 1997; Jost *et al*, 2000). HER2 was also expressed to significant levels while HER3 could not be detected. These results were confirmed by real-time quantitative RT-PCR analysis (Figure 1B).

### The HER-kinase pathway is functional in primary human keratinocytes and can be inhibited by gefitinib or cetuximab but not with pertuzumab

**Figure 2:**
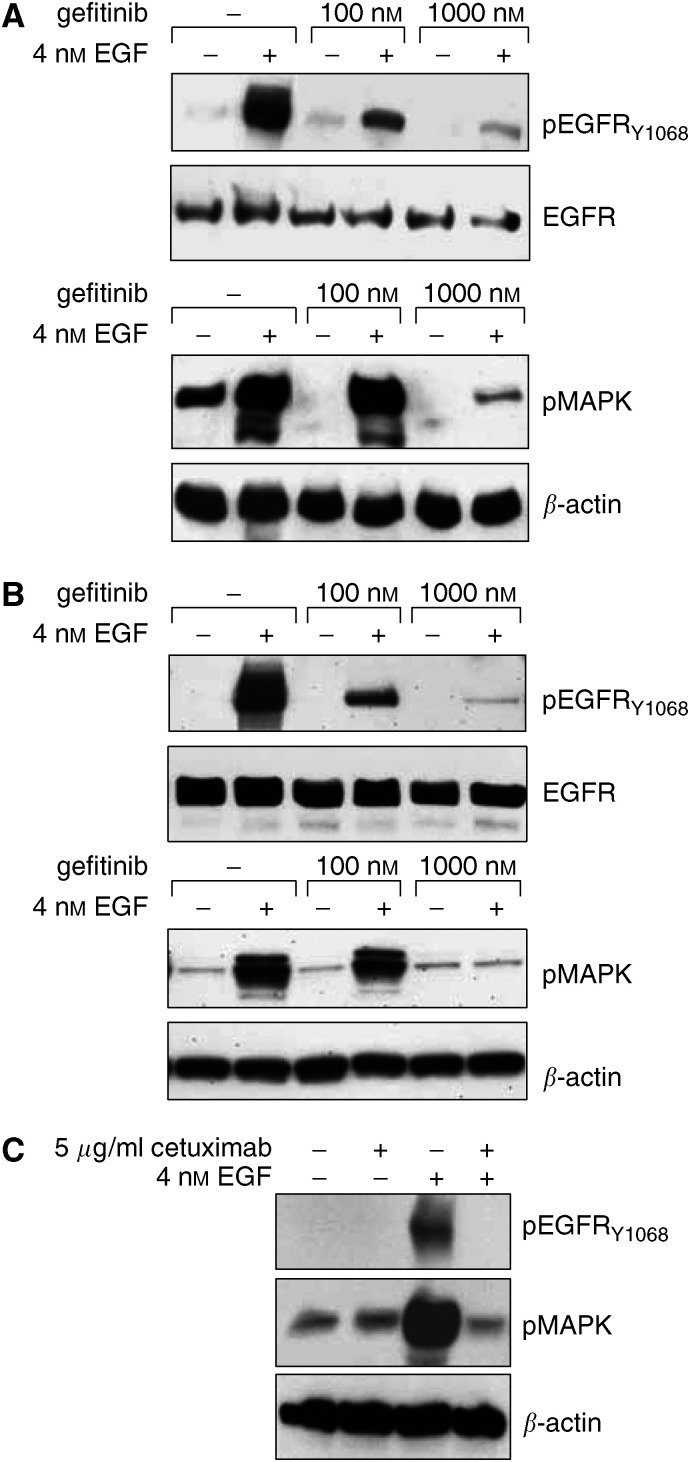
Effect of gefitinib and cetuximab on primary human keratinocytes. Representative immunoblots of lysates from NHEK (**A**, **C**) and 22Rv1 cells (**B**) are shown. (**A**) NHEK and (**B**) 22Rv1 cells were serum-starved for 24 h and treated with increasing levels of gefitinib 2 h before stimulation with 4 nM EGF for 10 min at 37°C. (**C**) NHEK were serum-starved for 24 hrs in the absence or presence of 5 *μ*g ml^−1^ cetuximab before stimulation with 4 nM EGF for 10 min at 37°C. Following stimulation cells were lysed in RIPA buffer and equal amounts of total protein were subjected to SDS-PAGE and transferred to PDVF-membranes. Blots were analyzed with specific antibodies to pEGFR, EGFR, pMAPK as indicated and reprobed for *β*-actin to account for loading differences.

We examined the dose escalating effect of gefitinib treatment on EGFR and MAPK phosphorylation after ligand stimulation since these phosphorylated proteins are functional surrogates for an activated HER signaling pathway (Albanell *et al*, 2001). EGF was able to stimulate EGFR and MAPK phosphorylation in NHEK that could be inhibited by gefitinib in a dose-dependent manner (Figure 2A). A lower concentration of gefitinib blocked ligand stimulated EGFR phosphorylation in human keratinocytes, whereas significant inhibition of downstream signaling, that is, MAPK phosphorylation, was achieved only at higher doses of gefitinib. The inhibitory concentrations of gefitinib in NHEK were similar to those observed for blocking pEGFR and pMAPK in 22Rv1 cells (Figure 2B), an androgen-independent prostate cancer cell line (Sramkoski *et al*, 1999). This confirms previously reported observations of differential inhibition of EGFR and MAPK phosphorylation in glioma cell lines (Li *et al*, 2003) and shows the effectiveness of gefitinib against EGFR signaling in human keratinocytes. To compare the effect of gefitinib with an anti-EGFR antibody, NHEK were treated with cetuximab, an anti-EGFR humanized chimeric mouse monoclonal antibody, which competitively inhibits receptor binding of EGF and other EGFR ligands (Mendelsohn, 2001). Figure 2C demonstrates that cetuximab was able to completely block [p]EGFR and [p]MAPK in primary human keratinocytes. Taken together, these results demonstrate that in primary human keratinocytes, EGF-dependent HER-receptor signaling can be specifically and completely blocked by EGFR-targeted therapies.

**Figure 3:**
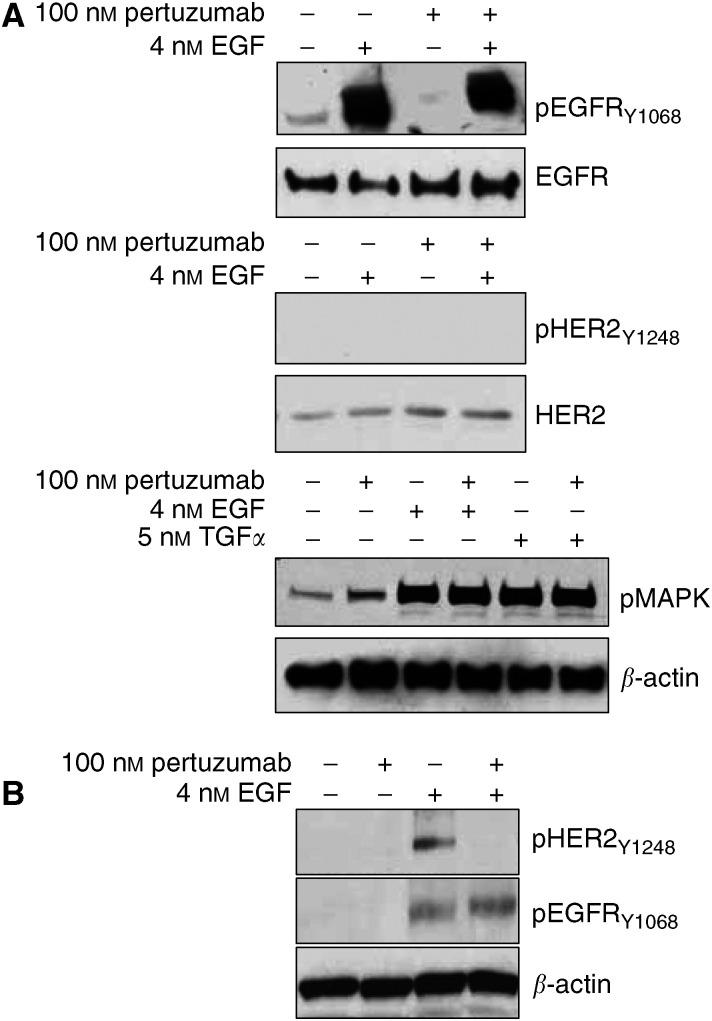
Effect of 2C4 on primary human keratinocytes. Representative immunoblots of lysates from NHEK (**A**) and 22Rv1 (**B**) cells are shown. Cells were starved in serum-free media for 24 h and treated with or without 100 nM pertuzumab 2 h before stimulation with 4 nM EGF or 5 nM TGF*α* for 10 min at 37°C. Cells were lysed and equal amounts of protein lysate were subjected to SDS-PAGE, transferred to PDVF-membranes and analyzed by using specific antibodies to EGFR, pEGFR, HER2 and pHER2, pMAPK. Blots were reprobed for *β*-actin to account for loading differences.

Unlike gefitinib and cetuximab, pertuzumab, which is a HER2-specific antibody that interferes with its heterodimerization with other receptor members, was unable to block EGF stimulated EGFR or MAPK phosphorylations in NHEK (Figure 3A). EGF was unable to induce HER2 phosphorylation in NHEK (Figure 3A) suggesting against the presence of a functional HER2 receptor in primary human keratinocytes even though HER2 protein is expressed. A parallel analysis of 22Rv1 cells demonstrated an activation of EGFR as well as HER2 in response to EGF, which could be inhibited by pertuzumab (Figure 3B). This suggests the formation of EGFR/HER2 heterodimers after EGF stimulation in 22Rv1 cells, but not in NHEK, since ligand-dependent activation of HER-kinase receptors is known to occur through the formation of receptor dimers (Yarden and Sliwkowski, 2001). These results are in accordance with the observation that pertuzumab inhibits HER2-containing heterodimers (Agus *et al*, 2002; Mendoza *et al*, 2002) and further implies that NHEK lack the formation of such heterodimers in response to EGF.

### HER2 does not interact with EGFR in primary human keratinocytes

To more specifically interrogate the HER-receptor dimerization status in primary human keratinocytes, we determined the presence of HER-kinase dimers in NHEK and 22Rv1 cells using coimmunoprecipitations and the *eTag*™ System. *eTag*™ Technology developed by ACLARA BioSciences is a multiplexed, solution phase analysis of proteins and nucleic acids. The multiplexed analysis is based on a set of electrophoresis-tag reporters (*eTag* reporters) – low molecular weight, fluorescent molecules with a unique and defined mobility in capillary electrophoresis (CE). The protein analysis is an antibody-based proximity assay that quantitatively measures protein–protein interactions (Chan-Hui *et al*, 2004; Salimi-Moosavi *et al*, 2004). *eTag* reporters are covalently linked to antibodies that specifically recognize the target protein. When bound to its target the *eTag* reporter is specifically cleaved and analyzed by capillary electrophoresis. The amount of free *eTag* reporter generated in the assay is proportional to the quantity of target protein (Miller, 2002). The *eTag*™ System can detect and quantify as few as several hundred receptors per cell and exceeds the precision and accuracy of Western blotting. More importantly, the *eTag*™ System allows for the evaluation of homodimer presence which typically cannot be interrogated with traditional coimmunoprecipitation assays.

EGFR coimmunoprecipitation experiments performed after EGF stimulation failed to demonstrate an EGFR/HER2 interaction in NHEK (data not shown). The lack of EGF-induced HER2 phosphorylation further argues against the presence of such heteromers. However, in 22Rv1 cells, EGF was able to induce HER2 phosphorylation that could be specifically blocked by pertuzumab (Figure 3B) suggesting the presence of EGFR/HER2 heterodimers. Probably due to assay detection limits we were unable to demonstrate this interaction in 22Rv1 cells using a coimmunoprecipitation assay.

**Figure 4:**
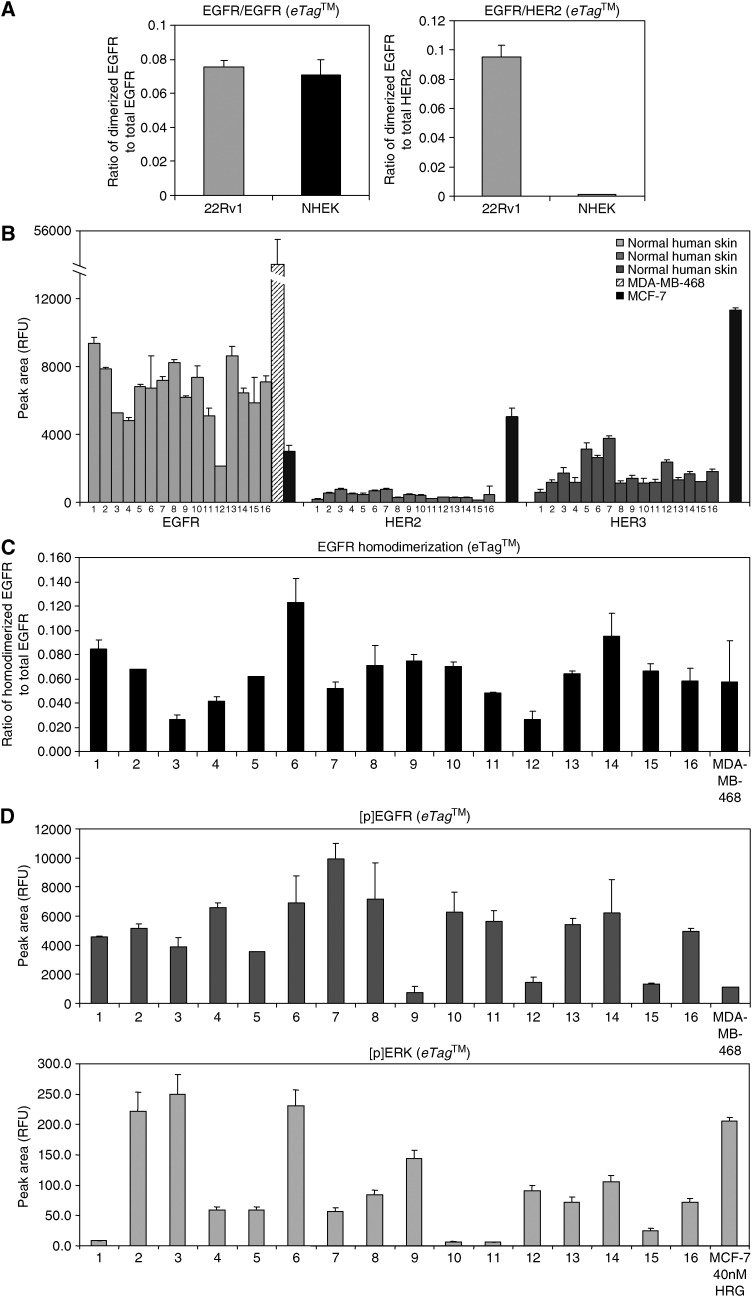
EGFR homodimers are the preferred dimerization form in normal human skin. NHEK and 22Rv1 cell lysates as well as FFPE human tissue samples and FFPE cancer cell lines were analyzed for total HER-receptor expression, phosphorylation, dimerization and ERK activation using *eTag*™ assays. (**A**) Comparison of EGFR homodimerization and EGFR/HER2 heterodimerization in NHEK and 22R1 cells. Data are presented as the ratio of dimerized EGFR to total EGFR and dimerized EGFR to total HER2 in each sample, respectively. (**B**) HER-kinase receptor expression in 16 normal human skin specimen (1–16) in comparison to MDA-MB-468 (17) and MCF-7 cells (18). Each bar represents the average relative fluorescence unit (RFU) of a triplicate analysis for each sample. (**C**) EGFR homodimerization in normal human skin samples (1–16) and MDA-MB-468 cells. EGFR homodimers are presented as the ratio of dimerized EGFR to total EGFR in each sample. Each bar represents the average value for a triplicate analysis for each sample. (**D**) EGFR phosphorylation and ERK activation in normal human skin specimen (1–16), MDA-MB-468 cells and MCF-7 cells. Data are presented as relative fluorescence units (RFU) for phosphorylated EGFR and phosphorylated ERK, respectively. Each bar represents an average value of a triplicate analysis for each sample.

Interestingly, in a parallel set of samples from 22Rv1 cells EGFR/HER2 heterodimers could be unequivocally detected using the *eTag*™-based assay (Figure 4A). This demonstrated the presence of EGFR/HER2 heterodimers after EGF stimulation as well as the sensitivity of this assay. Further, appreciable levels of EGFR homodimers were also detected after EGF stimulation in these cells. Using the *eTag*™ – assay after EGF induction in NHEK led to the detection of only EGFR homodimers (Figure 4A). This explains the lack of EGF-induced HER2 phosphorylation in these cells. The analysis demonstrated a preference for EGFR homodimerization and the absence of EGFR/HER2 heterodimerization in keratinocytes.

### EGFR homodimers are the predominant dimerization form in human skin

The *eTag*™ System allows the analysis of protein-protein interactions in FFPE clinical samples (Dua *et al*, 2004). Utilizing this assay, we studied the expression and dimerization status of HER-kinase receptors in 16 paraffin-embedded tissue sections of normal human skin obtained from patients with solid tumours. Figure 4B represents the expression levels of EGFR, HER2 and HER3 in these samples with MDA-MB-468 and MCF-7 cells as positive controls. EGFR, HER2 and HER3 were expressed in all 16 skin samples. The relative expression levels of various receptors in a given tissue section, represented by relative fluorescence units (RFU), cannot be directly compared to each other due to potential affinity and avidity differences between EGFR, HER2 and HER3 antibodies attached to distinct *eTag* reporters.

EGFR homodimers were detected in all 16 skin samples (Figure 4C), whereas EGFR/HER2 or HER2/HER3 heterodimers could not be found (data not shown). HER2 heterodimers are either absent in human skin or exist below the detection limit of this sensitive assay. All skin specimens also demonstrated EGFR and MAPK phosphorylation (Figure 4D) indicating a functional signaling pathway as a consequence of EGFR homodimerization. HER2 phosphorylation was undetectable in all skin specimens (data not shown). The EGFR overexpressing breast cancer cell line MDA-MB-468 forms EGFR homodimers in response to ligand stimulation. Similarly abundant levels of EGFR homodimers were measured in all skin samples.

## DISCUSSION

Our study provides a molecular rationale for the skin rash commonly observed with EGFR-targeted therapies (such as gefitinib and cetuximab) but not with therapies that target the HER2 receptor (such as pertuzumab and Herceptin). Although the reason for this discrepancy is unknown, the occurrence of skin rash has been of particular clinical relevance since several studies have correlated it with antitumour activity (Cohen *et al*, 2003; Saltz *et al*, 2003; Perez-Soler *et al*, 2004).

Using a sensitive and quantitative *eTag*™ dimerization assay we demonstrate that EGFR homodimers are the predominant isoform in human keratinocytes, while little or no HER2 heterodimers are found. These results are in disagreement with a previous study (Marques *et al*, 1999) that reported the presence of EGFR/HER2, EGFR/HER3 and HER2/HER3 heterodimers in the human keratinocyte cell line, HaCaT. However, their technique of choice, coimmunoprecipitation assay, is unsuited to detect homodimers. Another study has reported the absence of EGFR/HER2 heterodimers in mouse keratinocytes using coimmunoprecipitation assays (Xian *et al*, 1997). Perhaps the reason for such conflicting results has been the absence of a sensitive and quantitative assay to unambiguously measure receptor dimers *in vivo*.

The HER signaling network functions via productive signaling, that can only occur after the formation of receptor dimers. Our study demonstrates the absence of a functional target for a HER2-directed therapy, such as pertuzumab, in human skin due to the lack of detectable levels of HER2 heterodimers in human keratinocytes. Therefore, the absence of skin rash with HER2-directed therapies is not surprising. The functional consequences of EGFR homodimers and the lack of EGFR/HER2 heterodimers in primary human keratinocytes were demonstrated by two observations. First, gefitinib and cetuximab directly interfered with EGFR signaling by blocking EGFR and MAPK phosphorylation while pertuzumab did not. Second, HER2, despite considerable expression levels in keratinocytes and normal skin biopsies, did not engage into productive heterodimerization complexes as shown by the lack of HER2 phosphorylation, coimmunoprecipitation and *eTag*™ dimerization assays. Skin rash occurs when EGFR signaling is blocked with either antibodies or small molecule inhibitors. The antibody pertuzumab might not cause skin rash since HER2 does not seem to form any detectable functional HER2 heterodimers in human skin.

There are several speculations as to why HER2 fails to engage into productive dimerization complexes in human skin even though it has been suggested as a preferred heterodimerization partner (Tzahar *et al*, 1996; Graus-Porta *et al*, 1997; Hendriks *et al*, 2003). First, physical separation of EGFR and HER2 within the skin cells might be responsible for preventing their association (Stoll *et al*, 2001). EGFR is primarily expressed in the basal layer of the keratinocytes while HER2 and HER3 are expressed in the upper spinous layers (Piepkorn *et al*, 2003). A second possibility could be the functional separation of the two receptor subtypes. EGFR plays a key role during keratinocyte proliferation while HER2 is more important during the differentiation process (De Potter *et al*, 2001). Therefore, it is possible that EGFR and HER2 are not activated enough at the same time to cofunction in HER signaling. Third, studies that have challenged the concept of HER2 as a preferred dimerization partner, demonstrate that EGFR homodimerization and EGFR/HER2 heterodimerization occur at comparable affinities but HER2 has to be expressed at a certain threshold level to engage into productive EGFR/HER2 complexes (Hendriks *et al*, 2003). Given that HER2 protein is expressed at lower levels than EGFR in the human skin, it is possible that it is unable to achieve the minimal threshold to function as a coreceptor.

Our results suggest that the HER receptor dimer distribution may be different in the skin compared to a solid tumour within the same patient. It would be interesting to explore the levels of HER-receptor dimers within the skin and solid tumours of patients receiving HER-kinase directed therapies to address the question of HER-receptor dimer discordance. We could not directly demonstrate this discrepancy due to the lack of availability of tissue from paired skin and tumour biopsies. However, the skin samples analyzed in this study were obtained from patients with breast cancer. All 16 skin specimens only demonstrated the presence of EGFR homodimers and it is unlikely that the HER receptor dimer distribution would be identical in all 16 tumours. Interestingly, a recent study (Tan *et al*, 2004) described skin rash in 61% of 15 patients with metastatic breast cancer treated with erlotinib even though 14 of the 15 tumours were EGFR negative and had no objective clinical responses. This analysis of paired skin and tumour tissue clearly supports our results and questions the utility of skin rash as an optimal surrogate outcome marker for EGFR-directed therapies. However, skin may still be an effective pharmacodynamic surrogate for EGFR targeted drug activity. Other studies correlated the occurrence and the grade of the skin rash after HER-kinase therapies with clinical outcome, but again this concordance was not present in all studies (Baselga, 2003; Clark *et al*, 2003; Cohen *et al*, 2003; Saltz *et al*, 2003; van Zandwijk, 2003). Here, we provide a potential molecular explanation for this discrepancy. Severity of skin rash most likely relates to the number of EGFR homodimers in the skin and may not always represent the HER-kinase signaling status in a coexisting tumour.
